# The impact of nontransparent health communication during the COVID-19 pandemic on vaccine-hesitant people’s perception of vaccines

**DOI:** 10.3389/fpubh.2023.1256829

**Published:** 2024-01-08

**Authors:** Odette Wegwarth, Ralph Hertwig, Helge Giese, Harvey V. Fineberg

**Affiliations:** ^1^Heisenberg Chair for Medical Risk Literacy and Evidence-Based Decisions, Clinic for Anesthesiology and Intensive Care Medicine, Charité – Universitätsmedizin Berlin, Berlin, Germany; ^2^Center for Adaptive Rationality, Max Planck Institute for Human Development, Berlin, Germany; ^3^Gordon and Betty Moore Foundation, Palo Alto, CA, United States

**Keywords:** risk communication, public health communication, COVID-19 vaccine, vaccine hesitancy, evidence-based health communication, health literacy

## Abstract

**Background:**

Although transparency is crucial for building public trust, public health communication during the COVID-19 pandemic was often nontransparent.

**Methods:**

In a cross-sectional online study with COVID-19 vaccine-hesitant German residents (*N* = 763), we explored the impact of COVID-19 public health communication on the attitudes of vaccine-hesitant individuals toward vaccines as well as their perceptions of incomprehensible and incomplete information. We also investigated whether specific formats of public health messaging were perceived as more trustworthy.

**Results:**

Of the 763 participants, 90 (11.8%) said they had become more open-minded toward vaccines in general, 408 (53.5%) reported no change, and 265 (34.7%) said they had become more skeptical as a result of public health communication on COVID-19 vaccines. These subgroups differed in how incomprehensible they found public health communication and whether they thought information had been missing. Participants’ ranking of trustworthy public health messaging did not provide clear-cut results: the *fully transparent* message, which reported the benefit and harms in terms of absolute risk, and the *nontransparent* message, which reported only the benefit in terms of relative risk were both considered equally trustworthy (*p* = 0.848).

**Discussion:**

Increased skepticism about vaccines during the COVID-19 pandemic may have partly been fueled by subpar public health communication. Given the importance of public trust for coping with future health crises, public health communicators should ensure that their messaging is clear and transparent.

## 1 Introduction

Truthfulness, coherence, and transparency are essential attributes to evidence-based public health communication, and integral for building and maintaining public trust (1–5). During the COVID-19 pandemic, however, public health communicators sometimes neglected these attributes when explaining the dynamic course of the pandemic or the effectiveness of vaccines (1). For instance, shortly after vaccines became available, Anthony Fauci, the chief medical advisor to the U.S. president, suggested that herd immunity would be reached when around 60–70% of the population was vaccinated. He later raised the estimate to 70–75%, then to 75%, then to 80%, and finally to 85%, explaining that his original statements had not exactly reflected what he had considered to be true at the time: Instead, he had shifted the goal posts to accommodate what he felt the public would accept and to “nudge” people’s vaccination behavior (6). The concern here is not that public health leaders struggled to pass on the scientific uncertainty inherent in much of the pandemic or misunderstood the science behind the COVID-19 vaccine. Rather, the problem is that they strategically misrepresented their beliefs when communicating with the public, aiming to persuade rather than to inform. The underlying question is, however, whether scientific and political advisors should tailor their messages in order to persuade the public to follow recommended health measures, or whether they should accurately, fully, and transparently inform the public about the knowns and unknowns. According to evidence-based guidelines (7), transparency in health communication requires full disclosure of both the benefits and the harms of an intervention, provided in terms of absolute risk and accompanied by a denominator; it should also acknowledge any scientific uncertainty (8). Yet these standards are rarely met—nontransparency in health communication is a common problem in the relation between patients and public health experts (2, 8–13). Omissions of relevant information [e.g., of actual magnitude, harm, or denominator (14–17)] or misrepresentations [e.g., spurious precision, neglect of uncertainty (8, 14)] are frequent problems that, due to the nature of the pandemic, also arose repeatedly in public health communication during the COVID-19 pandemic. The use of nontransparent reporting techniques may be owed to the unprecedented dilemma politicians and public health experts faced: balancing transparent and truthful communication with the potential negative consequences of disclosing harms (1, 8, 18, 19). Nontransparency, however, carries both short- and long-term risks. In the short term, it may backfire and make people more, not less, reluctant to comply with public health measures. In the long term, incomplete or nontransparent information decreases trust in health authorities (20) and can foster the spread of conspiracy beliefs (1, 19). Some research communities therefore called for full transparent disclosure of COVID-19 information—including degrees of scientific uncertainties and the potential limitations of vaccines (8, 21, 22)—arguing that the negative impact on uptake caused by a public discovery of obfuscated facts post-rollout could be far worse than any negative impact arising from transparent communication upfront about negative or complicating factors (23). Vaccine-hesitant people—characterized by a strong need for information on both benefits and harms and a potential willingness to get vaccinated if information is convincing (19, 24)—can be especially deterred by nontransparent messaging.

The objectives of our study were threefold: to examine the impact of COVID-19 public health communication on vaccine-related attitudes among individuals who were hesitant toward COVID-19 vaccination, to identify elements of COVID-19 public health communication that were perceived as incomprehensible or lacking, and to assess the perceived trustworthiness of vaccine-related messages.

## 2 Materials and methods

### 2.1 Study design

Between December 2022 and February 2023, we conducted a cross-sectional online survey study with a national sample of 763 COVID-19 vaccine-hesitant German residents aged ≥18 years (Table 1). Participants were drawn from a well-established probability-based internet panel maintained by the survey sampling institute respond (Cologne, Germany). No official statistics are available on the gender, age, ethnicity, and education of COVID-19 vaccine-hesitant individuals in Germany, so a quota sampling method to gain representativeness regarding demographics was not possible. Data presented within this manuscript were retrieved as a supplementary poll on subjective risk communication perception during the second survey wave of a larger vaccine-hesitancy project, for which the sample size calculation and the inclusion and exclusion criteria can be found elsewhere (19). The vaccine-hesitancy project was approved by the Institutional Review Board (IRB) of the Charité – Universitätsmedizin Berlin (EA1/360/21). Written informed consent was obtained online from all participants at the outset of the study.

**Table 1:** Overview on sufficiency of provided information within each of the four health messages in accordance with the guidelines of evidence-based health communication.

	Deceptive message	Nontransparent message	Partially transparent message	Fully transparent message
*Description of benefits*				
Relative risks ➔ usually provide large numbers (e.g., 90%), but are intransparent due to no provision of the denominator	**X**	**X**		
Absolute risks ➔ usually provide small numbers (e.g., 1 less in 1,000 people) and are transparent formats due to the provision of the denominator			**X**	**X**
*Description of harms*				
Relative risks ➔ usually provide large numbers (e.g., 90%), but are intransparent due to no provision of the denominator				
Absolute risks ➔ usually provide small numbers (e.g., 1 less in 1,000 people) and are transparent formats due to the provision of the denominator	**X**			**X**
*Overall assessment of the reporting of benefits and harms*				
Mismatched framing ➔ communicating benefits as relative risks (appear large) and harms as absolute risks (small), most misleading communication technique	**X**			
Insufficient ➔ either information on benefit or on harms is missing		**X**	**X**	
Sufficient ➔ information on both benefits and harms are given				**X**

### 2.2 Survey questionnaire

We aimed to identify aspects of public health communication on COVID-19 that could cause unintended negative changes in people’s general attitudes about vaccines. Specifically, we probed which information people considered unintelligible and/or inconsistent and which information they had wanted but did not receive, and what kind of vaccine-related messages they would consider trustworthy. Statements used to explore this matter were drawn from common public statements made by politicians and scientists (e.g., “90% effectiveness of the vaccine”) during the pandemic. They were selected based on their deviation from and omission of essential information required by the guidelines for evidence-based health information (5).

To assess changes in participants’ vaccine-related attitudes, we first asked whether over the course of the pandemic they had become more open-minded, become more skeptical, or remained unchanged in their opinion toward vaccines in response to the communication efforts of politicians and scientists. Response options were offered in randomized order.

We next asked participants whether they perceived the communication of politicians and scientists as incomprehensible during the pandemic (for exact wording of questions, see Supplementary material). If participants responded in the affirmative, they were shown a set of six statements: five descriptions of nontransparent communication that were discussed in the media and by the general public during the pandemic (e.g., “Politicians and/or scientists claimed things about COVID-19 and the vaccine which then did not occur.”) and the option “none of these reasons.” Participants selected what they considered the three most relevant statements and ranked them according to their relevance (1 = highest rank). Next, we asked whether participants thought that any specific information had been missing in COVID-19 communication. If they responded in the affirmative, they saw another set of six statements: five descriptions of what may have been missing from the perspective of evidence-based health communication (e.g., age-adjusted information on the benefit–harm ratio of the vaccine) and the option “none of these reasons.” Participants selected and ranked the top three statements in order of importance (1 = highest rank). Finally, to investigate which format of communication would be perceived as most trustworthy, participants were presented with four messages. All four explained the effectiveness of the vaccine but the messages differed in the degree to which they conformed to the principles of evidence-based health communication that have been established in a national guideline on this matter (7)—that is, using absolute numbers for both benefits and harms and adjusting to the same denominator for both outcomes. Based on these principles, messages presented either only the benefit of the vaccine or both the benefit and the potential harms of the vaccine, and presented information either in terms of relative risk (e.g., 90% effectiveness) and/or in terms of absolute risk (e.g., reduce risk of infection from XX to YY in 1,000).

The *deceptive* message (see Table 1), the most problematic of the four, described the benefit in terms of relative risk and the in terms of absolute risk, a highly misleading technique known as “mismatched framing.” The *nontransparent* message, which was modeled after the most common approach during the pandemic, reported only the benefit in terms of relative risk (90% effectiveness). The *partially transparent* message reported only the benefit in terms of absolute risk. Finally, the *fully transparent* message, which is aligned with the principles of effective evidence-based health communication, described both benefit and harms in terms of absolute risk. Participants were then asked to rank the messages according to their perceived trustworthiness (1 = highest rank/most perceived trustworthiness). The exact wording for each of the questions and the messages can be found in Supplementary material.

### 2.3 Analysis

We provide summary statistics as proportions with a Clopper-Pearson 95% confidence interval (CI) for binary and Goodman CI for multinomial proportions. To understand what aspects participants considered most incomprehensible and what information they felt was particularly absent from public health communication, we selected and analyzed the proportion of the highest ranking given to each of the descriptions of incomprehensible communication and missing information. Chi-square one-sample tests were used to assess whether a uniform distribution of values was present, followed by binomial tests to compare highest rank proportion across reasons. Chi-square tests were used to test for differences between groups (with 2,000 replicates simulated Fisher-exact test in case of too-small cell sizes). Multinomial logistic regression analyses were used to investigate the associations between change in attitude toward vaccines and participant demographics (age, gender, education). *p* values were 2-sided, if possible, with statistical significance set at *p* < 0.05 for single comparisons. For multiple comparisons, statistical significance was set after Bonferroni correction at *p* < 0.003 for comparing the six statements regarding incomprehensible communication or missing information, at *p* < 0.010 for comparing differences between the four messages, and at *p* < 0.013 for comparing differences between the three subgroups. All data were stored and analyzed utilizing R basic software and the packages DescTools, nnet, and car.

## 3 Results

### 3.1 Participants

Of the 763 participants (Table 2), 652 (85.5%) were younger than 60 years old and 413 (54.1%) were female. Since the first wave of the study (April–May, 2022), 74 (9.7%) said they had been vaccinated against COVID-19 and had not experienced a COVID-19 infection, 56 (7.3%) had been vaccinated and had experienced a COVID-19 infection, 200 (26.2%) had not been vaccinated and had experienced a COVID-19 infection, and 433 (56.7%) reported having been neither vaccinated nor infected.

**Table 2:** Summary of demographics.

	Sample size *N* = 763
Age 18–34 years 35–59 years ≥ 60 years	Number (%)^a^ 193 (25.3) 459 (60.2) 111 (14.5)
Gender Female Male Other	Number (%)^a^ 347 (45.5) 413 (54.1) 3 (0.4)
Education No qualifications/no information provided Basic/intermediate College-entry level qualification Higher education degree	Number (%)^a^ 13 (1.7) 310 (40.7) 222 (29.1) 218 (28.6)
COVID-19 vaccination since 1st wave Yes	Number (%) 130 (17.0)
COVID-19 infection since 1st wave Yes	Number (%) 256 (33.6)
Healthcare professional No	Number (%) 701 (91.9)

^a^Percentages are rounded and may not add up to 100.

### 3.2 Change in attitudes toward vaccines in general

Ninety participants (11.8%; 95% CI: 9.3–14.9) said they had become more open-minded toward vaccines in general, 408 participants (53.5, 95% CI: 49.1–57.8) reported no change in their attitude, and 265 participants (34.7%; 95% CI: 30.7–39.0) said they had become more skeptical over the course of the pandemic in response to the communication of scientists and politicians.

### 3.3 Incomprehensible public health communication

Of the 265 participants who had become more skeptical toward vaccines, 91.3% (*n* = 242; 95% CI: 87.3–94.4) indicated that they found the scientific and political communication incomprehensible during the pandemic, as did 77.2% (*n* = 315; 95% CI: 72.8–81.2) of the 408 who reported no attitude change and 25.6% (*n* = 23; 95% CI: 16.9–35.4) of the 90 who became more open-minded. The extent to which the subgroups considered that communication incomprehensible differed significantly overall [χ^2^(2, *N* = 763) = 160.06, *p* < 0.001, *V* = 0.458], and also when comparing each group with the others (all *p* < 0.001).

**Figure 1:**
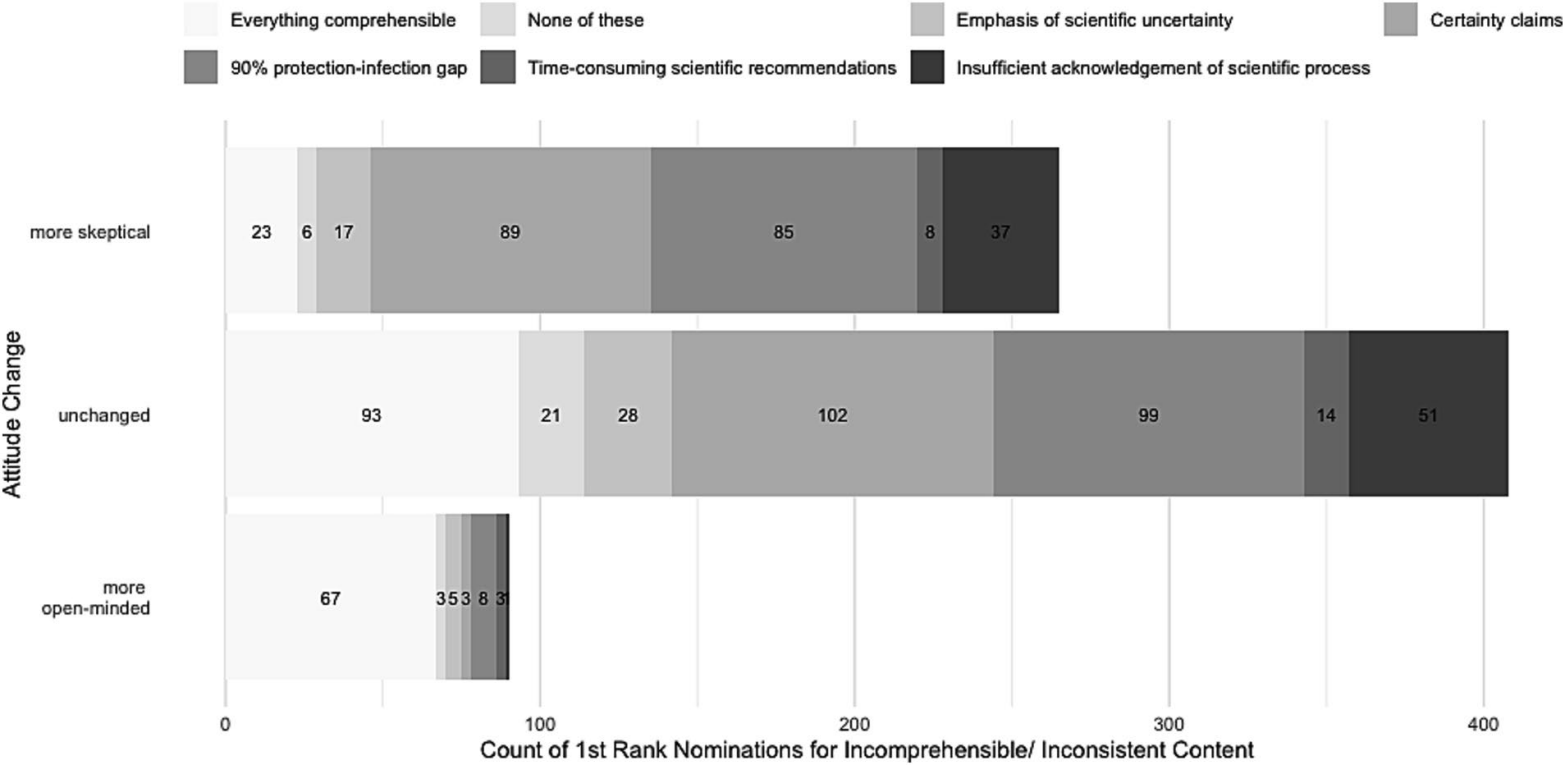
Distribution of absolute number of participants’ highest rankings of reasons for incomprehensibility in public health communication.

Subgroups also differed on how they ranked reasons that communication was perceived as incomprehensible [χ^2^(10, *N* = 580) = 23.81, *p*_Fisher_ = 0.008, *V* = 0.143]. Most of the participants whose skepticism had increased (*n* = 89; 36.8, 95% CI: 29.1–45.2) gave the highest ranking to a perceived discrepancy between the claims of politicians and scientists and the perceived reality of the pandemic, followed by a perceived discrepancy between the communicated 90% vaccine effectiveness and their observations of more people who got infected despite being vaccinated (Omicron effect; *n* = 85; 35.1 95% CI: 27.5–43.5). These two reasons did not differ significantly in their proportion of highest rankings (*p* = 0.820) but differed from the other remaining four options, which received a significantly lower proportion of highest rankings (*p* < 0.001). We found a similar pattern for participants who reported no change in their vaccine-related attitude [χ^2^(5, *N* = 557) = 7.40, *p* = 0.192, *V* = 0.115]: The same two reasons most often garnered the highest ranking (claims of politicians and scientists not matching the reality of the situation: 32.4%, *n* = 102, 95% CI: 25.9–39.7; discrepancy between the communicated 90% vaccine effectiveness and observed infection rates: 31.4%, *n* = 99, 95% CI: 25.0–38.7; *p* = 0.888) and the ranking of these two reasons was significantly higher than for the remaining four options (all *p* < 0.001). Of participants who had become more open-minded, the greatest number gave the highest ranking to the discrepancy between the communicated 90% vaccine effectiveness and the actual infection rates among vaccinated people (34.8%, *n* = 8; 95% CI:15.1–61.5), followed by 21.7% (*n* = 5, 95% CI: 7.4–49.2) who took issue with politicians’ and scientists’ accounts of uncertainty and unpredictability around the COVID-19 pandemic, but sample sizes of this subgroup were too small to permit statistically meaningful inferences. Figure 1 shows the order of proportion of the highest rankings across all options per subgroup.

### 3.4 Missing information in public health communication

A total of 61.1% (*n* = 162/265; 95% CI: 55.0–67.0) of participants who had become more skeptical, 36.7% of those who had become more open-minded (*n* = 33/90; 95% CI: 26.8–47.5), and 35.5% of those without change (*n* = 145/408; 95% CI: 30.9–40.4) thought certain information had been missing from scientific and political health communication around COVID-19. The extent to which participants thought health information had been missing did not differ between those who had become more open-minded and those whose attitude did not change (*p* = 0.840); however, participants who had become more skeptical reported missing information significantly more often than the other two subgroups (all *p* < 0.001).

**Figure 2:**
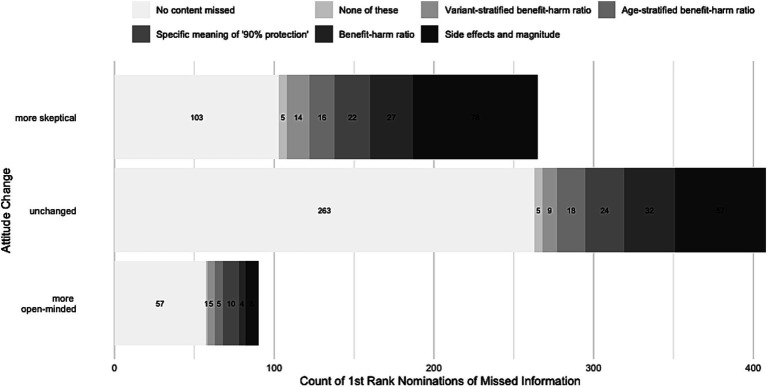
Distribution of absolute number of participants’ highest rankings of missing aspects in public health communication.

When asked about what information participants felt was missing in COVID-19 communication of politicians and scientists, most participants gave the highest ranking to detailed and numerical information on vaccine side effects (*n* = 143; 42.1%; 95% CI: 35.2–49.2). All other reasons received significantly lower proportions of highest rankings (all *p* < 0.001; e.g., “numerical information on the benefit-to-harm ratio” [*n* = 63; 18.5%; 95% CI: 13.6–24.7]; “details on what the statement of a 90% protection exactly refers to” [*n* = 56; 16.5%; 95% CI: 11.8–22.4]). Details on the proportion of the highest ranking across all options can be seen in Figure 2. Notably, highest rankings of missed information did not differ significantly between subgroups [χ^2^(10, *N* = 340) = 14.42, *p*_Fisher_ = 0.308, *V* = 0.146].

### 3.5 Trustworthy messaging formats

Across all participants, the *fully transparent* message, which reported the benefit and harms in terms of absolute risk (28.3%, *n* = 216, 95% CI: 24.4–32.5), and the *nontransparent* message, which reported only the benefit in terms of relative risk (29.0%, *n* = 221, 95% CI: 25.0–33.2), were considered the most trustworthy formats (*p* = 0.848). Both messages were considered significantly more trustworthy than the *deceptive* message, which reported the benefit as relative risk and harms as absolute risks; 19.1%, *n* = 146, 95% CI: 15.8–22.9; all *p* < 0.001), but were not seen as significantly more trustworthy than the *partially transparent* message, which reported the benefit in terms of absolute risk only (23.6%, *n* = 180, 95% CI:20.0–27.6; *p* > 0.046). The nomination pattern for the most trustworthy format did not systematically differ across subgroups [χ^2^(6, *N* = 763) = 7.14, *p* = 0.308, *V* = 0.068].

### 3.6 Association between change in attitude and demographics

In multinomial regression analysis, becoming more skeptical toward vaccines due to COVID-19 communication of politicians and scientists was not significantly associated with any demographic variables, including age, gender, and education (all *p* > 0.279). Becoming more open-minded, however, was significantly (two-thirds) lower among those 60 years of age or older [χ^2^(2) = 9.79, *p* = 0.007; odds ratio (*OR*): 0.303, 95% CI: 0.106–0.860, *p* = 0.025], and was significantly higher (doubled) among those with a college-entry level of education [χ^2^(2) = 7.82, *p* = 0.020; *OR*: 1.99, 95% CI: 1.20–3.30, *p* = 0.007]. Gender did not systematically affect the subgroups [χ^2^(2) = 3.34, *p* = 0.188].

## 4 Discussion

In the midst of a rapidly evolving pandemic, shortcomings in communication about risks and uncertainties may have undermined some people’s trust in health guidance. Of 763 COVID-19 vaccine-hesitant people, roughly a third reported that scientists’ and politicians’ communication on COVID-19 had raised their skepticism toward vaccines. Of those, nearly all attributed their skepticism to incomprehensible communication, and most indicated that they thought key information had been missing—including information about uncertainties, harms, and magnitude, and up-to-date estimates on COVID-19 vaccines’ effectiveness. These concerns about missing information also applied to participants who reported no attitude change and, to a much lesser extent, to participants who reported that they had become more open-minded toward vaccines.

These findings highlight that effective vaccines in themselves are not sufficient to address a public health crisis. Truthful and intelligible messaging characterized by transparent absolute effects and the acknowledgment of uncertainties is necessary to maintain public confidence in and motivation toward vaccination. Public health communication, however, does not always meet these standards of transparency. An investigation of more than 2,500 websites (25) from public health communicators such as the World Health Organization (WHO), the U.K.’s National Health Service (NHS), and the U.S. Centers for Disease Control (CDC) revealed that a majority of health behavior claims (84%) failed to report any effect size for the efficacy of a preventive behavior. Instead, websites simply offered long lists of negative health outcomes (e.g., 177 distinct claims about the negative effects of obesity) without any numbers (25). Only 1% of all claims made on these websites complied partially with the guidelines on evidence-based health communication by providing the magnitude of a preventive health behavior in absolute numbers. Communicators may choose to exclude effect sizes in order to keep things simple and to avoid overwhelming people (25, 26). However, the COVID-19 pandemic made apparent that the information asymmetry between experts and the public has shrunk considerably since the advent of the internet and the risk of losing public trust is substantial. Whatever privileged knowledge experts may once have possessed is now often publicly available online, and members of the public can easily examine evidence directly from the source (6). Inaccuracies or distortions of effect sizes and omission of key information or uncertainties are thus more likely to be detected and made public, resulting in a loss of trust in public health communicators.

In order to make health messages understandable, health care leaders can use insights from social sciences (27) and in addition format evidence in ways that readily convey important details about outcomes and uncertainties. For example, fact boxes provide a concise summary of scientific evidence by listing outcomes in a tabular structure. Outcomes are presented as absolute risks or frequencies for two distinct populations: one engaging in a specific behavior and the other abstaining from it. The tabular structure makes it easy to compare the benefits, risks, and uncertainties associated with each outcome, typically expressed as the number of individuals per 1,000 or 10,000; it also ensures that the evidence is clear and substantiated by ample supporting data (28, 29). Also, tested communication examples exist that help public health communicators to receptively explain scientific uncertainty to the public (8, 19). Especially people who are skeptical of the certitude conveyed by public officials have been found to be most responsive to an open communication about uncertainties (8, 19).

Participants rated the fully transparent format (reporting benefit and harms as absolute risk) as most trustworthy as often as the nontransparent format (reporting only the benefit as relative risk). Notably, the nontransparent format mirrored how politicians and scientists often communicated the COVID-19 vaccine’s effectiveness during the pandemic, with approximately a quarter of participants reporting that they had struggled to comprehend that public health message without more concrete numbers. In the same vein, among the 143 participants who ranked information on vaccine-related side effects and their magnitude highest when asked what information they felt had been missing, roughly a third chose the nontransparent message—which did not report any harms—as the most trustworthy one. One possible explanation for these seemingly contradictory findings might be the “mere-exposure effect” (or “familiarity effect”) (30): People tend to prefer things that they are exposed to repeatedly over novel things. Familiarity can also influence trust in other types of information; for example, repeated exposure to a brand’s message or logo enhances trust in the brand (31). While exposure alone is likely not sufficient to establish solid trust in information—other factors, such as credibility, expertise, and consistency also play a role—nevertheless the familiar nature of the nontransparent message may have influenced some of participants’ rankings. Another reason for the varied results on the most trustworthy format might be that participants felt that none of the messages, regardless of numerical transparency, adequately addressed the aspects that mattered to them. Consequently, they may have perceived all messages as untrustworthy. However, it is worth noting that bracketing the nontransparent scenario (which reported only the vaccine’s benefit with 90% effectiveness)—and thereby removing its potential advantage due to familiarity—results in the fully transparent message being perceived as most trustworthy. This message aligns most closely with evidence-based health reporting.

Our study has limitations. First, all participants exhibited hesitancy toward COVID-19 vaccination. As a result, these findings may not generalize to the broader population. Understanding effective communication strategies for vaccine-hesitant people is nevertheless crucial, as these individuals are also more likely to resist adopting preventive behaviors in a public health emergency. A second potential limitation is that participants had to choose from predetermined sets of statements. All statements were derived from public statement made by politicians and scientist (e.g., “90% effectiveness of vaccine”) during the pandemic and selected due to their violation of requirements of the guideline of evidence-based health information (5). Still, they may not have reflected all the aspects that individuals thought were incomprehensible or missing. However, only a small number of participants (less than 5% per subgroup) chose the none-of-these-reasons option, suggesting that the options provided were adequate. Third, due to the highly volatile pandemic situation, we did not validate this opinion survey. However, survey validation is typically more crucial when attempting to measure a psychological construct, such as competences or psychological traits, rather than opinions, as was the case with our survey. Finally, our online survey used forced-choice items, which has been linked with higher dropout rates. However, of the 798 participants who started the second wave of our survey, only 35 dropped out. The resulting 4.4% dropout rate is considerably lower than the dropout rates ranging from 20% to over 40% commonly observed in research on online surveys (32), indicating that the inclusion of forced-choice items did not significantly impact participants’ response behavior.

## 5 Conclusion

Communicating intelligibly, transparently, and in an unbiased manner about the impact of preventive behavior on health and longevity is a complex task. In some cases, the effects of preventive behavior on relevant health outcomes may not have been sufficiently assessed by current science. Furthermore, if insights rely on correlational studies or trials with small and nonrepresentative samples, it can be difficult to provide a good estimate of the causal effect of preventive behavior. However, if the scientific community is uncertain about causal effects, it has an ethical responsibility to transparently communicate this uncertainty to the public. Likewise, if risk estimates exist, public health communicators must avoid misleading techniques such as relative risks, mismatched framing, magnitude neglect, and upper-bound reporting.

Emphasizing only the benefits of a vaccine can undermine confidence in public health messages and public health communicators—especially when people’s own observations appear to contradict the message. The ability of the research and public health communities to effectively respond to future health challenges relies, in large part, on gaining and maintaining the trust of the general population. Trust is more likely to endure when institutions demonstrate transparency, competence, and reliability. Frank assessments of what individuals can and cannot expect from preventive behaviors and interventions may, over time, encourage more support for public health policies rather than less. In light of the abundance of information available today, politicians’ and scientists’ commitment to clarity and transparency is fundamental to cultivating the trust necessary for dealing with any future health crises.

## 6 Plain summary

Open and transparent communication about the benefits, the harms and the uncertainties surrounding the effectiveness of prevention—especially in situations of health emergencies—is essential to building public trust. During the corona (COVID-19) pandemic, however, public health communicators often neglected these standards when explaining the effectiveness of COVID-19 vaccines. Nontransparent communication carries short- and long-term risks though, including decreased trust in health authorities and the spread of conspiracy beliefs. The reported study investigated—to the best of our knowledge for the first time—the impact of the public health communication during the corona pandemic on COVID-19 hesitant people and their attitudes toward vaccines in general. Of the 763 COVID-19 vaccine-hesitant people surveyed, almost 35% reported that communication from scientists and politicians about COVID-19 had raised their skepticism about vaccines in general. Of these 265, about 91% attributed their skepticism to incomprehensible communication, and about 61% said that they felt that key information was missing—including information about uncertainties, harms, and magnitude of the COVID-19 vaccine. These findings highlight that effective vaccines alone are not sufficient to address a public health crisis. Truthful and intelligible messaging, with transparent absolute effects and the acknowledgment of uncertainties, is needed to maintain motivation for vaccination and public trust.

## Data availability statement

The datasets presented in this study can be found at an online repositories. The names of the repository/repositories and accession number(s) can be found at: Open Science Framework/OSF (https://osf.io/pu5vm/).

## Ethics statement

The studies involving humans were approved by the Institutional Ethics Review Board (IRB) of the Charité – Universitätsmedizin Berlin (EA1/360/21). The studies were conducted in accordance with the local legislation and institutional requirements. The participants provided their written informed consent to participate in this study.

## Author contributions

OW: Conceptualization, Data curation, Formal analysis, Funding acquisition, Investigation, Methodology, Project administration, Resources, Software, Supervision, Validation, Visualization, Writing – original draft, Writing – review & editing. RH: Conceptualization, Investigation, Methodology, Writing – review & editing. HG: Conceptualization, Data curation, Formal analysis, Investigation, Methodology, Visualization, Writing – review & editing. HF: Conceptualization, Investigation, Supervision, Writing – review & editing.
