# Supplementary material for: The impact of nontransparent health communication during the COVID-19 pandemic on vaccine-hesitant people’s perception of vaccines
Source: Front Public Health. 2024 Jan 8;11:1256829. doi: 10.3389/fpubh.2023.1256829 (PMC10800610; doi:10.3389/fpubh.2023.1256829)
Supplement: Supplementary file 1 [file Table_1.DOCX]

Supplementary Material

*Odette Wegwarth, Ralph Hertwig, Helge Giese, & Harvey V. Fineberg*: **The Impact of Nontransparent Health Communication During the COVID-19 Pandemic on Vaccine-Hesitant People’s Perception of Vaccines**

**Table of Content:**

1. **Survey Questions**

**Questionnaire Survey (Translated from German)**

1. Over the course of the COVID-19 pandemic, has your attitude towards vaccination in general become more open-minded, remained unchanged, or become more skeptical due to COVID-19 communication from politicians and/or scientists?

a. More open-minded

b. Unchanged

c. More skeptical

1. Have you found the communication by politicians and/or scientists about the COVID vaccine incomprehensible and/or inconsistent? [presented in random order]

a. Yes, I found the information incomprehensible and/or inconsistent.

b. No, I did not find the information incomprehensible and/or inconsistent.

[***IF YES***] What did you find incomprehensible and/or inconsistent? Please select up to three reasons and rank them in order of relevance to you (1, 2, 3). I found it incomprehensible and/or inconsistent that...

- - - - politicians and scientists emphasized the significance of science on one hand, but then disregarded it in practice (e.g., by preempting recommendations of the Standing Vaccination Committee/STIKO)
      - scientific committees such as STIKO required substantial time to formulate vaccination recommendations.
      - politicians and scientists stated that the vaccine offered protection of over 90%, but then a significant number of vaccinated individuals still contracted COVID-19.
      - politicians and scientists claimed things about COVID 19 and the vaccine, which then did not occur.
      - politicians and/or scientists emphasized the scientific uncertainties surrounding the vaccine and the impossibility of predicting exact future outcomes.
      - None of these reasons.

1. Did you feel that politicians and/or scientists did not provide enough information about the COVID-19 vaccine?

a. Yes, I felt that information was missing.

b. No, I did not feel felt that information was missing.

[***IF YES***] What information did you feel was missing? Please select up to three answers and rank them in order of importance to you (1, 2, 3). I was missing information about...

- - - 1. the side effects of the COVID-19 vaccine and their magnitude.
      2. the numerical ratio between the vaccine’s benefits and harms.
      3. the specific context und meaning of the statement, "90% protection through vaccination."
      4. whether the benefits and side effects of the vaccine would depend on age.
      5. whether the benefits and side effects of the vaccination would depend on the prevailing COVID-19 variant.
      6. None of these reasons.

1. We will now describe the effectiveness of the COVID-19 vaccine in different ways. Please evaluate the information in terms of trustworthiness. Please rank the statements from "1" (most trustworthy) to "4" (least trustworthy) [randomized presentation of statements].

| - **Statements on vaccine effectiveness** | - Trustworthiness of statement - (1 = most trustworthy / 4 = least trustworthy) |
| --- | --- |
| The probability of a COVID-19 infection is approximately 90% lower in individuals who are fully vaccinated compared to those who are not vaccinated. The risk of experiencing severe facial swelling, temporary facial paralysis, hives, or myocarditis/pericarditis due to vaccination is estimated to be between 0.1% and 0.01%. |  |
| The probability of a COVID-19 infection is approximately 90% lower in individuals who are fully vaccinated compared to those who are not vaccinated. |  |
| Out of 10,000 vaccinated individuals, approximately 2,500 will experience a COVID-19 infection within 4 months. The risk of severe facial swelling, temporary facial paralysis, or hives due to vaccination is less than one case per 10,000 people, while the risk of developing myocarditis or pericarditis is approximately one case per 10,000 people. In comparison, out of 10,000 unvaccinated individuals, around 4,000 will get an COVID-19 infection within 4 months. |  |
| Out of 10,000 vaccinated individuals, approximately 2,500 will experience a COVID-19 infection within 4 months. In comparison, out of 10,000 unvaccinated individuals, around 4,000 will get an COVID-19 infection within 4 months. |  |

**
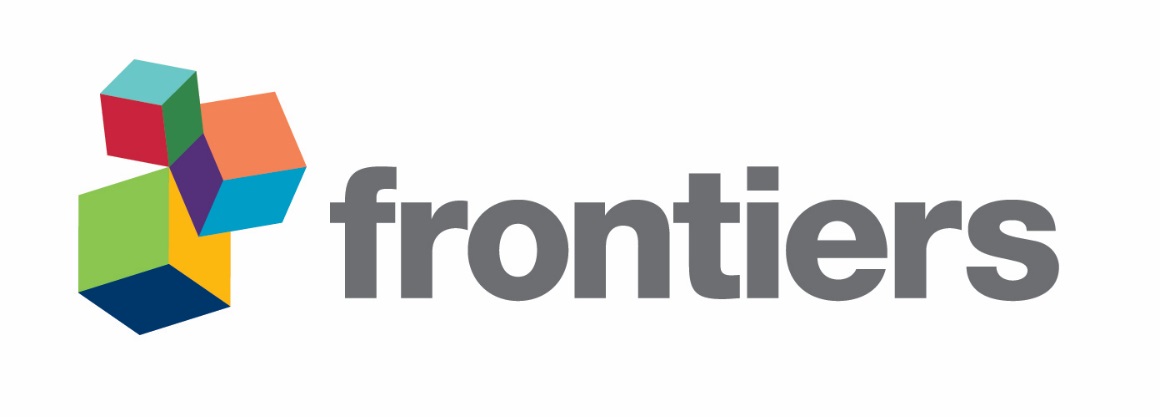
**
